# Supplementary material for: Hot spot prediction in protein-protein interactions by an ensemble system
Source: BMC Syst Biol. 2018 Dec 31;12(Suppl 9):132. doi: 10.1186/s12918-018-0665-8 (PMC6311905; doi:10.1186/s12918-018-0665-8)
Supplement: Supplementary file 2 — All relevant MATLAB code. A simple Matlab implement of our predictor. (ZIP 19,742 kb) [file 12918_2018_665_MOESM2_ESM.zip › Additional file 2/Read ASA/ASA website/ASEdb/1JTGA.htm]

 NetSurfP - Protein Surface Accessibility and Secondary Structure Predictions

  

|  |  |  |
| --- | --- | --- |
|  |  | NetSurfP - Protein Surface Accessibility and Secondary Structure Predictions  Technical University of Denmark |

  


---

```
# For publication of results, please cite:
# A generic method for assignment of reliability scores applied to solvent accessibility predictions.
# Bent Petersen, Thomas Nordahl Petersen, Pernille Andersen, Morten Nielsen and Claus Lundegaard
# BMC Structural Biology 2009, 9:51 doi:10.1186/1472-6807-9-51
#
# Column 1: Class assignment - B for buried or E for Exposed - Threshold: 25% exposure, but not based on RSA
# Column 2: Amino acid
# Column 3: Sequence name
# Column 4: Amino acid number
# Column 5: Relative Surface Accessibility - RSA
# Column 6: Absolute Surface Accessibility
# Column 7: Z-fit score for RSA prediction
# Column 8: Probability for Alpha-Helix
# Column 9: Probability for Beta-strand
# Column 10: Probability for Coil
E H  1JTGA                  1    0.853 155.124   0.341   0.000   0.001   0.999
E P  1JTGA                  2    0.500  70.978  -0.736   0.321   0.003   0.675
E E  1JTGA                  3    0.682 119.110   1.371   0.694   0.003   0.303
B T  1JTGA                  4    0.141  19.598   0.626   0.858   0.002   0.139
E L  1JTGA                  5    0.273  49.913   0.901   0.938   0.007   0.055
E V  1JTGA                  6    0.643  98.875   1.405   0.938   0.007   0.055
E K  1JTGA                  7    0.402  82.609   2.317   0.970   0.001   0.030
B V  1JTGA                  8    0.024   3.689   1.176   0.970   0.001   0.030
E K  1JTGA                  9    0.428  87.978   2.463   0.970   0.001   0.030
E D  1JTGA                 10    0.579  83.492   2.242   0.970   0.001   0.030
B A  1JTGA                 11    0.101  11.119   0.767   0.970   0.001   0.030
B E  1JTGA                 12    0.095  16.579  -0.895   0.923   0.002   0.076
E D  1JTGA                 13    0.719 103.550   1.149   0.858   0.002   0.139
E Q  1JTGA                 14    0.722 128.931   1.037   0.694   0.003   0.303
B L  1JTGA                 15    0.171  31.383   0.514   0.176   0.004   0.820
E G  1JTGA                 16    0.741  58.356   0.481   0.005   0.015   0.979
B A  1JTGA                 17    0.126  13.863  -0.461   0.005   0.045   0.951
E R  1JTGA                 18    0.365  83.562   1.340   0.004   0.616   0.381
B V  1JTGA                 19    0.083  12.772   0.975   0.001   0.959   0.040
B G  1JTGA                 20    0.029   2.298   0.166   0.001   0.959   0.040
B Y  1JTGA                 21    0.024   5.214   1.015   0.000   0.983   0.017
B I  1JTGA                 22    0.042   7.807   0.911   0.000   0.983   0.017
B E  1JTGA                 23    0.019   3.372   1.260   0.000   0.983   0.017
B L  1JTGA                 24    0.065  11.847   1.119   0.000   0.983   0.017
B D  1JTGA                 25    0.083  11.960   0.243   0.001   0.900   0.099
B L  1JTGA                 26    0.161  29.461  -0.133   0.005   0.262   0.733
E N  1JTGA                 27    0.660  96.565   0.264   0.004   0.085   0.910
E S  1JTGA                 28    0.414  48.509  -0.113   0.018   0.019   0.964
E G  1JTGA                 29    0.437  34.408  -1.382   0.018   0.019   0.964
E K  1JTGA                 30    0.439  90.220   0.948   0.021   0.451   0.528
E I  1JTGA                 31    0.290  53.705   1.109   0.011   0.918   0.071
B L  1JTGA                 32    0.107  19.628   1.298   0.011   0.918   0.071
B E  1JTGA                 33    0.207  36.233   0.302   0.011   0.918   0.071
B S  1JTGA                 34    0.173  20.299  -1.139   0.021   0.756   0.223
E F  1JTGA                 35    0.342  68.720   0.475   0.021   0.451   0.528
E R  1JTGA                 36    0.283  64.853   0.446   0.019   0.141   0.840
E P  1JTGA                 37    0.424  60.095  -1.348   0.018   0.047   0.935
E E  1JTGA                 38    0.638 111.476  -0.778   0.018   0.047   0.935
E E  1JTGA                 39    0.363  63.434   0.473   0.019   0.141   0.840
E R  1JTGA                 40    0.385  88.234   0.634   0.022   0.359   0.619
B F  1JTGA                 41    0.117  23.502   1.360   0.021   0.279   0.699
B P  1JTGA                 42    0.186  26.436   0.075   0.056   0.142   0.802
B M  1JTGA                 43    0.075  14.947  -1.298   0.268   0.043   0.689
B M  1JTGA                 44    0.027   5.363  -0.611   0.430   0.016   0.555
B S  1JTGA                 45    0.050   5.825  -1.268   0.717   0.014   0.269
B T  1JTGA                 46    0.049   6.796  -0.713   0.923   0.002   0.076
B F  1JTGA                 47    0.050   9.995   0.163   0.923   0.002   0.076
B K  1JTGA                 48    0.053  10.943   0.523   0.970   0.001   0.030
B V  1JTGA                 49    0.044   6.778   0.204   0.970   0.001   0.030
B L  1JTGA                 50    0.070  12.744   0.251   0.970   0.001   0.030
B L  1JTGA                 51    0.041   7.599   0.181   0.970   0.001   0.030
B C  1JTGA                 52    0.022   3.075   0.787   0.970   0.001   0.030
B G  1JTGA                 53    0.077   6.076   0.280   0.970   0.001   0.030
B A  1JTGA                 54    0.073   8.001   0.899   0.970   0.001   0.030
B V  1JTGA                 55    0.027   4.150   0.948   0.970   0.001   0.030
B L  1JTGA                 56    0.053   9.668   1.009   0.970   0.001   0.030
E S  1JTGA                 57    0.331  38.840   1.378   0.970   0.001   0.030
B R  1JTGA                 58    0.203  46.441   0.655   0.970   0.001   0.030
B I  1JTGA                 59    0.094  17.390   0.224   0.923   0.002   0.076
E D  1JTGA                 60    0.651  93.838   0.931   0.858   0.002   0.139
E A  1JTGA                 61    0.702  77.349   0.850   0.406   0.004   0.590
E G  1JTGA                 62    0.555  43.647  -0.808   0.053   0.005   0.942
E Q  1JTGA                 63    0.723 129.110   0.312   0.018   0.019   0.964
B E  1JTGA                 64    0.141  24.580  -0.201   0.005   0.015   0.979
E Q  1JTGA                 65    0.515  91.961   1.027   0.005   0.015   0.979
B L  1JTGA                 66    0.180  32.921  -0.032   0.018   0.047   0.935
E G  1JTGA                 67    0.628  49.416  -0.225   0.018   0.047   0.935
E R  1JTGA                 68    0.398  91.050   0.510   0.005   0.336   0.660
E R  1JTGA                 69    0.437 100.165   1.291   0.002   0.816   0.182
B I  1JTGA                 70    0.065  11.988   0.950   0.001   0.900   0.099
E H  1JTGA                 71    0.484  87.967   1.539   0.001   0.900   0.099
B Y  1JTGA                 72    0.077  16.348   0.879   0.002   0.816   0.182
E S  1JTGA                 73    0.312  36.543   1.164   0.022   0.552   0.426
E Q  1JTGA                 74    0.601 107.374   0.204   0.125   0.227   0.648
E N  1JTGA                 75    0.617  90.270   0.261   0.191   0.086   0.723
B D  1JTGA                 76    0.183  26.327  -1.022   0.199   0.152   0.649
B L  1JTGA                 77    0.273  49.913  -0.384   0.216   0.235   0.548
E V  1JTGA                 78    0.506  77.788  -0.734   0.135   0.317   0.548
E E  1JTGA                 79    0.546  95.334  -0.790   0.064   0.216   0.721
E Y  1JTGA                 80    0.530 113.218  -2.273   0.018   0.088   0.893
B S  1JTGA                 81    0.325  38.090  -2.173   0.018   0.047   0.935
E P  1JTGA                 82    0.316  44.897  -1.992   0.019   0.141   0.840
E V  1JTGA                 83    0.364  55.870  -0.622   0.022   0.359   0.619
B T  1JTGA                 84    0.276  38.337  -0.859   0.021   0.451   0.528
E E  1JTGA                 85    0.406  70.963  -1.541   0.022   0.359   0.619
E K  1JTGA                 86    0.311  64.034  -0.299   0.021   0.279   0.699
E H  1JTGA                 87    0.459  83.492   0.194   0.019   0.141   0.840
E L  1JTGA                 88    0.415  75.913  -0.562   0.018   0.047   0.935
E T  1JTGA                 89    0.693  96.064  -1.532   0.018   0.019   0.964
E D  1JTGA                 90    0.490  70.609  -0.145   0.053   0.043   0.903
E G  1JTGA                 91    0.532  41.845   0.910   0.064   0.216   0.721
B M  1JTGA                 92    0.086  17.269   1.558   0.064   0.216   0.721
B T  1JTGA                 93    0.177  24.536   1.487   0.191   0.086   0.723
B V  1JTGA                 94    0.037   5.748   0.306   0.975   0.003   0.022
E R  1JTGA                 95    0.286  65.402   1.341   0.970   0.001   0.030
B E  1JTGA                 96    0.178  31.149   0.841   0.988   0.000   0.012
B L  1JTGA                 97    0.019   3.534   1.434   0.988   0.000   0.012
B C  1JTGA                 98    0.026   3.707   1.035   0.988   0.000   0.012
B S  1JTGA                 99    0.249  29.171   1.385   0.988   0.000   0.012
B A  1JTGA                100    0.078   8.540  -0.016   0.988   0.000   0.012
B A  1JTGA                101    0.023   2.579   0.923   0.970   0.001   0.030
B I  1JTGA                102    0.039   7.234   0.292   0.975   0.003   0.022
B T  1JTGA                103    0.136  18.808   0.778   0.938   0.007   0.055
E M  1JTGA                104    0.249  49.765   0.514   0.802   0.014   0.185
B S  1JTGA                105    0.035   4.114  -1.250   0.339   0.016   0.645
B D  1JTGA                106    0.170  24.540  -1.208   0.176   0.004   0.820
B N  1JTGA                107    0.082  11.990  -0.952   0.600   0.003   0.397
B T  1JTGA                108    0.199  27.643   0.320   0.970   0.001   0.030
B A  1JTGA                109    0.023   2.535   0.795   0.970   0.001   0.030
B A  1JTGA                110    0.019   2.050   1.061   0.970   0.001   0.030
B N  1JTGA                111    0.103  15.050   0.359   0.988   0.000   0.012
B L  1JTGA                112    0.088  16.168   1.384   0.988   0.000   0.012
B L  1JTGA                113    0.023   4.266   1.009   0.988   0.000   0.012
B L  1JTGA                114    0.042   7.709   0.198   0.970   0.001   0.030
E T  1JTGA                115    0.403  55.952   0.845   0.923   0.002   0.076
E T  1JTGA                116    0.283  39.280   0.625   0.858   0.002   0.139
B I  1JTGA                117    0.094  17.334   0.115   0.406   0.004   0.590
B G  1JTGA                118    0.340  26.750  -1.278   0.053   0.005   0.942
B G  1JTGA                119    0.262  20.604  -0.785   0.053   0.005   0.942
B P  1JTGA                120    0.252  35.759   0.573   0.782   0.003   0.216
E K  1JTGA                121    0.638 131.257   0.558   0.970   0.001   0.030
E E  1JTGA                122    0.373  65.076   1.435   0.970   0.001   0.030
B L  1JTGA                123    0.025   4.541   1.022   0.970   0.001   0.030
B T  1JTGA                124    0.147  20.444   1.302   0.970   0.001   0.030
E A  1JTGA                125    0.533  58.748   1.649   0.988   0.000   0.012
B F  1JTGA                126    0.225  45.218   1.833   0.988   0.000   0.012
B L  1JTGA                127    0.020   3.662   0.929   0.970   0.001   0.030
E H  1JTGA                128    0.485  88.258   1.802   0.970   0.001   0.030
E N  1JTGA                129    0.682  99.801   1.660   0.923   0.002   0.076
B M  1JTGA                130    0.147  29.335   1.430   0.600   0.003   0.397
E G  1JTGA                131    0.449  35.360  -0.563   0.053   0.005   0.942
B D  1JTGA                132    0.090  12.926  -0.131   0.016   0.005   0.979
E H  1JTGA                133    0.585 106.448   0.365   0.018   0.088   0.893
E V  1JTGA                134    0.410  63.063  -0.330   0.020   0.205   0.775
B T  1JTGA                135    0.074  10.278  -0.161   0.022   0.359   0.619
E R  1JTGA                136    0.292  66.982   0.753   0.022   0.552   0.426
B L  1JTGA                137    0.073  13.366  -0.682   0.021   0.451   0.528
E D  1JTGA                138    0.338  48.763  -0.015   0.021   0.279   0.699
E R  1JTGA                139    0.366  83.814  -0.470   0.056   0.142   0.802
E W  1JTGA                140    0.309  74.315  -1.043   0.056   0.142   0.802
E E  1JTGA                141    0.341  59.590  -1.232   0.118   0.150   0.732
E P  1JTGA                142    0.317  44.926  -1.879   0.113   0.087   0.800
E E  1JTGA                143    0.343  59.975  -1.221   0.184   0.043   0.773
B L  1JTGA                144    0.203  37.169  -1.459   0.184   0.043   0.773
B N  1JTGA                145    0.182  26.659  -1.401   0.113   0.043   0.844
E E  1JTGA                146    0.581 101.553  -1.642   0.113   0.043   0.844
E A  1JTGA                147    0.366  40.377  -2.306   0.058   0.017   0.925
E I  1JTGA                148    0.361  66.877  -1.296   0.018   0.019   0.964
E P  1JTGA                149    0.379  53.780  -1.619   0.018   0.019   0.964
E N  1JTGA                150    0.551  80.725  -2.192   0.003   0.003   0.994
E D  1JTGA                151    0.412  59.441  -1.143   0.003   0.003   0.994
E E  1JTGA                152    0.436  76.134  -1.555   0.016   0.005   0.979
E R  1JTGA                153    0.454 103.943   0.256   0.016   0.005   0.979
B D  1JTGA                154    0.138  19.915  -1.362   0.018   0.019   0.964
B T  1JTGA                155    0.135  18.683   0.506   0.052   0.084   0.864
B T  1JTGA                156    0.025   3.454  -0.188   0.052   0.084   0.864
B M  1JTGA                157    0.042   8.344   0.610   0.257   0.016   0.727
B P  1JTGA                158    0.018   2.611   0.940   0.970   0.001   0.030
B V  1JTGA                159    0.174  26.667   0.900   0.970   0.001   0.030
B A  1JTGA                160    0.058   6.370  -0.660   0.970   0.001   0.030
B M  1JTGA                161    0.017   3.482   1.391   0.970   0.001   0.030
B A  1JTGA                162    0.019   2.116   0.779   0.970   0.001   0.030
E T  1JTGA                163    0.227  31.513   1.934   0.970   0.001   0.030
B T  1JTGA                164    0.031   4.327   0.168   0.970   0.001   0.030
B L  1JTGA                165    0.022   3.937   1.060   0.970   0.001   0.030
B R  1JTGA                166    0.187  42.800   0.773   0.970   0.001   0.030
B K  1JTGA                167    0.180  37.026   1.128   0.970   0.001   0.030
B L  1JTGA                168    0.026   4.724   0.639   0.970   0.001   0.030
B L  1JTGA                169    0.118  21.661   0.706   0.923   0.002   0.076
E T  1JTGA                170    0.508  70.501   0.903   0.600   0.003   0.397
E G  1JTGA                171    0.502  39.515   0.228   0.176   0.004   0.820
E E  1JTGA                172    0.668 116.682   0.030   0.053   0.005   0.942
B L  1JTGA                173    0.223  40.905  -1.053   0.058   0.017   0.925
B L  1JTGA                174    0.091  16.625   0.011   0.018   0.019   0.964
E T  1JTGA                175    0.422  58.573   1.175   0.018   0.019   0.964
E L  1JTGA                176    0.583 106.729   0.338   0.923   0.002   0.076
E A  1JTGA                177    0.631  69.580   0.623   0.923   0.002   0.076
B S  1JTGA                178    0.106  12.435  -0.889   0.970   0.001   0.030
B R  1JTGA                179    0.124  28.304   0.163   0.988   0.000   0.012
E Q  1JTGA                180    0.551  98.462   0.993   0.988   0.000   0.012
E Q  1JTGA                181    0.281  50.169   1.888   0.970   0.001   0.030
B L  1JTGA                182    0.022   4.028   0.582   0.970   0.001   0.030
B I  1JTGA                183    0.199  36.852   1.028   0.970   0.001   0.030
E D  1JTGA                184    0.511  73.664   0.862   0.970   0.001   0.030
B W  1JTGA                185    0.102  24.531   0.430   0.923   0.002   0.076
B M  1JTGA                186    0.065  12.966  -0.695   0.858   0.002   0.139
E E  1JTGA                187    0.409  71.400   0.101   0.782   0.003   0.216
E A  1JTGA                188    0.598  65.911  -0.391   0.522   0.016   0.462
B D  1JTGA                189    0.207  29.800  -1.170   0.181   0.016   0.803
E K  1JTGA                190    0.429  88.184  -1.038   0.113   0.087   0.800
B V  1JTGA                191    0.269  41.361  -1.546   0.052   0.084   0.864
E A  1JTGA                192    0.332  36.619  -0.955   0.184   0.043   0.773
E G  1JTGA                193    0.446  35.116  -0.857   0.181   0.016   0.803
E P  1JTGA                194    0.471  66.849  -0.243   0.339   0.016   0.645
B L  1JTGA                195    0.155  28.472  -0.076   0.354   0.048   0.598
B L  1JTGA                196    0.045   8.331  -0.226   0.354   0.048   0.598
E R  1JTGA                197    0.351  80.287   0.061   0.354   0.048   0.598
E S  1JTGA                198    0.493  57.768  -1.326   0.268   0.043   0.689
B A  1JTGA                199    0.272  30.018  -1.664   0.257   0.016   0.727
B L  1JTGA                200    0.141  25.799   0.054   0.115   0.016   0.868
B P  1JTGA                201    0.238  33.829  -1.191   0.113   0.043   0.844
E A  1JTGA                202    0.663  73.007  -0.958   0.058   0.017   0.925
E G  1JTGA                203    0.596  46.921  -1.170   0.018   0.019   0.964
B W  1JTGA                204    0.078  18.735   0.356   0.018   0.088   0.893
E F  1JTGA                205    0.255  51.078   0.390   0.021   0.451   0.528
B I  1JTGA                206    0.064  11.877   0.529   0.021   0.756   0.223
B A  1JTGA                207    0.027   2.942  -1.433   0.023   0.655   0.322
B D  1JTGA                208    0.089  12.810  -0.681   0.022   0.552   0.426
B K  1JTGA                209    0.053  10.902  -0.675   0.022   0.359   0.619
B S  1JTGA                210    0.069   8.052  -0.654   0.021   0.279   0.699
B G  1JTGA                211    0.064   5.013  -1.691   0.021   0.279   0.699
B A  1JTGA                212    0.234  25.776  -0.835   0.064   0.216   0.721
B G  1JTGA                213    0.142  11.152  -0.100   0.113   0.087   0.800
E E  1JTGA                214    0.535  93.517  -0.900   0.184   0.043   0.773
E R  1JTGA                215    0.476 108.935  -0.087   0.181   0.016   0.803
B G  1JTGA                216    0.189  14.898  -1.180   0.113   0.043   0.844
B S  1JTGA                217    0.148  17.357  -0.105   0.064   0.216   0.721
B R  1JTGA                218    0.200  45.708   0.407   0.022   0.552   0.426
B G  1JTGA                219    0.028   2.188   0.128   0.002   0.816   0.182
B I  1JTGA                220    0.033   6.160   0.508   0.001   0.900   0.099
B I  1JTGA                221    0.022   4.051   0.374   0.001   0.959   0.040
B A  1JTGA                222    0.017   1.906   0.931   0.001   0.959   0.040
B A  1JTGA                223    0.034   3.703   0.298   0.000   0.983   0.017
B L  1JTGA                224    0.025   4.523   0.119   0.001   0.959   0.040
B G  1JTGA                225    0.263  20.682   1.173   0.001   0.959   0.040
E P  1JTGA                226    0.288  40.938   1.046   0.004   0.420   0.576
E D  1JTGA                227    0.777 112.023  -0.170   0.005   0.015   0.979
E G  1JTGA                228    0.823  64.754  -0.561   0.003   0.003   0.994
E K  1JTGA                229    0.356  73.250   0.539   0.005   0.045   0.951
E P  1JTGA                230    0.285  40.385   1.177   0.004   0.514   0.481
B S  1JTGA                231    0.106  12.376   1.523   0.001   0.900   0.099
B R  1JTGA                232    0.028   6.435   1.150   0.001   0.959   0.040
B I  1JTGA                233    0.019   3.552   1.317   0.000   0.983   0.017
B V  1JTGA                234    0.012   1.783   1.821   0.000   0.983   0.017
B V  1JTGA                235    0.013   1.921   1.562   0.000   0.983   0.017
B I  1JTGA                236    0.018   3.256   1.372   0.001   0.959   0.040
B Y  1JTGA                237    0.058  12.395   0.120   0.001   0.959   0.040
B T  1JTGA                238    0.052   7.268  -0.188   0.004   0.514   0.481
E T  1JTGA                239    0.453  62.873  -0.238   0.005   0.045   0.951
E G  1JTGA                240    0.490  38.594  -0.454   0.003   0.003   0.994
E S  1JTGA                241    0.495  57.979  -1.612   0.005   0.015   0.979
E Q  1JTGA                242    0.662 118.269  -0.593   0.016   0.005   0.979
E A  1JTGA                243    0.478  52.687  -1.043   0.016   0.005   0.979
E T  1JTGA                244    0.470  65.189   0.406   0.053   0.005   0.942
E M  1JTGA                245    0.325  64.932  -0.020   0.858   0.002   0.139
E D  1JTGA                246    0.662  95.423   1.227   0.970   0.001   0.030
E E  1JTGA                247    0.414  72.308   1.045   0.923   0.002   0.076
B R  1JTGA                248    0.066  15.160  -0.786   0.970   0.001   0.030
E N  1JTGA                249    0.320  46.877   0.461   0.970   0.001   0.030
E R  1JTGA                250    0.530 121.256   2.057   0.970   0.001   0.030
B Q  1JTGA                251    0.094  16.699   1.452   0.970   0.001   0.030
B I  1JTGA                252    0.021   3.922   1.549   0.970   0.001   0.030
E A  1JTGA                253    0.285  31.429   1.563   0.970   0.001   0.030
E E  1JTGA                254    0.465  81.288   1.835   0.970   0.001   0.030
B I  1JTGA                255    0.036   6.568   0.354   0.970   0.001   0.030
B G  1JTGA                256    0.032   2.542   0.021   0.988   0.000   0.012
E A  1JTGA                257    0.396  43.683   1.974   0.988   0.000   0.012
B S  1JTGA                258    0.241  28.198   1.657   0.970   0.001   0.030
B L  1JTGA                259    0.024   4.358   0.602   0.970   0.001   0.030
B I  1JTGA                260    0.156  28.842   0.520   0.923   0.002   0.076
E K  1JTGA                261    0.676 138.991   1.555   0.858   0.002   0.139
E H  1JTGA                262    0.412  74.943   1.108   0.694   0.003   0.303
E W  1JTGA                263    0.350  84.151  -1.494   0.053   0.005   0.942

Explain the output.  Go back.
```

---
